# Limited Oxidative Stress Favors Resistance to Skeletal Muscle Atrophy in Hibernating Brown Bears (*Ursus Arctos*)

**DOI:** 10.3390/antiox8090334

**Published:** 2019-08-22

**Authors:** Blandine Chazarin, Anna Ziemianin, Alina L. Evans, Emmanuelle Meugnier, Emmanuelle Loizon, Isabelle Chery, Jon M. Arnemo, Jon E. Swenson, Guillemette Gauquelin-Koch, Chantal Simon, Stéphane Blanc, Etienne Lefai, Fabrice Bertile

**Affiliations:** 1 Université de Strasbourg, CNRS, IPHC UMR 7178, F-670000 Strasbourg, France; 2 Centre National d’Etudes Spatiales, CNES, F-75001 Paris, France; 3 Department of Forestry and Wildlife Management, Inland Norway University of Applied Sciences, Campus Evenstad, NO-2480 Koppang, Norway; 4 CarMen Laboratory, INSERM 1060, INRA 1397, University of Lyon, F-69600 Oullins, France; 5 Department of Wildlife, Fish, and Environmental Studies, Swedish University of Agricultural Sciences, SE-901 83 Umeå, Sweden; 6 Faculty of Environmental Sciences and Natural Resource Management, Norwegian University of Life Sciences, NO-1432 Ås, Norway; 7 Norwegian Institute for Nature Research, NO-7485 Trondheim, Norway; 8 Université d’Auvergne, INRA, UNH UMR1019, F-63122 Saint-Genès Champanelle, France

**Keywords:** hibernation, brown bears, skeletal muscle, cold response, oxidative stress, NRF2

## Abstract

Oxidative stress, which is believed to promote muscle atrophy, has been reported to occur in a few hibernators. However, hibernating bears exhibit efficient energy savings and muscle protein sparing, despite long-term physical inactivity and fasting. We hypothesized that the regulation of the oxidant/antioxidant balance and oxidative stress could favor skeletal muscle maintenance in hibernating brown bears. We showed that increased expressions of cold-inducible proteins CIRBP and RBM3 could favor muscle mass maintenance and alleviate oxidative stress during hibernation. Downregulation of the subunits of the mitochondrial electron transfer chain complexes I, II, and III, and antioxidant enzymes, possibly due to the reduced mitochondrial content, indicated a possible reduction of the production of reactive oxygen species in the hibernating muscle. Concomitantly, the upregulation of cytosolic antioxidant systems, under the control of the transcription factor NRF2, and the maintenance of the GSH/GSSG ratio suggested that bear skeletal muscle is not under a significant oxidative insult during hibernation. Accordingly, lower levels of oxidative damage were recorded in hibernating bear skeletal muscles. These results identify mechanisms by which limited oxidative stress may underlie the resistance to skeletal muscle atrophy in hibernating brown bears. They may constitute therapeutic targets for the treatment of human muscle atrophy.

## 1. Introduction

Hibernation is a natural strategy allowing certain mammals to spare energy and survive harsh cold winters with limited food supply [1,2]. During hibernation, the significant drop in animal’s metabolic rate, reduction in heart and respiratory rates, decreased body core temperature, and physical inactivity are key mechanisms that underlie reduced energy utilization [1]. Hence, despite food deprivation, hibernation can be used for weeks to months depending on how much energy animals have stored during the pre-hibernation period [3]. In a number of hibernators, periodic arousals occur during hibernation and are believed to be necessary returns to euthermy for reversing adverse effects of torpor. Indeed, hibernation/torpor triggers physiological costs, including sleep deprivation [4], decline of synaptic contacts and dendritic branching [5], memory impairment [6], reduced immunocompetence [7], compromised circulation and ionic balance [8], and oxidative stress [1].

Oxidative stress is defined as an imbalance that favors oxidant production over antioxidant protection and leads to disruption in redox signaling and to molecular damage in cells [9]. Aerobic metabolism implies generation of reactive oxygen species (ROS), the main ones being superoxide anion (O_2_•^−^), hydroxyl radical (•OH), and hydrogen peroxide (H_2_O_2_). At low or moderate concentration, ROS play physiological roles, e.g., in immunity [10]. However, ROS are unstable compounds, and when they are present in excess, they may damage biomolecules like nucleic acids, proteins, and lipids. Ultimately, these damages can induce a loss of function and cause cell death [10]. Several antioxidant systems fight oxidative stress in cells, including the glutathione system and antioxidant enzymes to deactivate ROS, and machineries for repair and elimination of damaged molecules [9,11]. Oxidative stress is triggered in relation with a number of conditions, including for example immune cell activation, inflammation, ischemia, infection, cancer, mental stress, food deprivation, and aging [10,12,13,14]. In skeletal muscles, oxidative stress has been reported to be induced in various disease states [15,16], but also by exercise [17], microgravity conditions [18], fasting [19], and muscle disuse [20]. In these latter examples, oxidative stress has been directly connected to the promotion of muscle proteolysis and inhibition of protein synthesis, thereby contributing to muscle atrophy [20].

The occurrence of oxidative stress during hibernation is a complex and still not fully resolved question. As stated above, hibernation in mammals is most often characterized by periodic arousals, which implies a rapid increase in body temperature and rise in oxygen consumption that may be responsible for increased ROS production. During deep torpor bouts, one can hypothesize that ROS production may either decrease due to low body temperatures and reduced activity of mitochondrial respiration, or increase due to specific alteration of electron transfer at mitochondrial ROS-producing sites. Focusing on steady-state torpor compared to the euthermic active period, oxidative stress has been shown to be induced in the intestinal mucosa of hibernating 13-lined ground squirrels (*Ictidomys tridecemlineatus*) [21], and in red blood cells of the hibernating American black bear (*Ursus americanus*) [22]. An increase in the levels of H_2_O_2_ in the brain and heart, and of malondialdehyde (a product of lipid peroxidation) in the brain of Daurian ground squirrels (*Spermophilus dauricus*) has been found during hibernation, while antioxidant enzyme expression and/or activity was decreased in the heart, brain, and plasma [23]. Other studies have shown upregulation of some antioxidants during hibernation, supposedly to confer resistance to oxidative stress, like in the brown adipose tissue and liver of hibernating ground squirrels (*Spermophilus citellus*) [24], and in the heart and brain of several species of hibernating bats (*Myotis lucifugus*, *Roussetus leschenaultia*, *Cynopterus sphinx*, and *Myotis ricketti*) [25,26]. In hibernating *I. tridecemlineatus*, a few studies have observed upregulation of antioxidants (expression and/or activity) in the liver, kidney, heart, skeletal muscle, and brown and white adipose tissues [27,28,29,30,31,32,33], whereas other studies showed no change in the brain, heart and liver tissues [34,35]. In skeletal muscle of hibernating *I. tridecemlineatus*, it has also been reported that ROS production is decreased [33], and that there is no evidence of oxidative stress [32]. Finally, oxidative stress has been found to be unchanged in the liver but decreased in the brain and, to a lower extent, the brown adipose tissue, from hibernating arctic ground squirrels (*Spermophilus parryii*) [36]. Hence, the regulation of the oxidant/antioxidant balance and oxidative stress appears to largely differ according to the species and tissues considered.

In bears, very few results have been reported concerning oxidative stress during hibernation [22]. By moving to a carbohydrate-rich diet in autumn [37], Swedish brown bears (*Ursus arctos*) accumulate large amounts of fat stores that allow for 6–7 months of hibernation [3,38], during which inactive animals do not eat, drink, urinate, defecate, or exhibit arousal episodes [39,40]. Strikingly, bears lose a very moderate amount of muscle protein during hibernation [41,42]. It could be hypothesized that the regulation of the oxidant/antioxidant balance and oxidative stress is such that it favors the maintenance of skeletal muscle in hibernating bears. Indeed, bears’ body temperature drops by only a few degrees during hibernation [39,43]. This implies that the 75% decrease in metabolic rate that has been recorded in hibernating bears is essentially achieved via active metabolic inhibition, independent of body temperature [44]. The body temperature of hibernating bears (32–34 °C) is also the temperature at which expression levels of cold-inducible proteins CIRBP and RBM3 [45] is maximal, as shown in murine fibroblasts [46], murine organotypic hippocampal slice cultures [47], and human cortical neurons [48]. Accordingly, RBM3 has been reported to be upregulated in skeletal muscle of hibernating black bears [49]. Alleviation of oxidative stress has been attributed to CIRBP in mouse testes, hepatocytes, the neuroblastoma cell line neuro2a, and in rat cortical neurons [50,51,52,53], and to RBM3 in human SH-SY5Y neuroblastoma cells and C_2_C_12_ mouse myoblasts [54,55]. Therefore, the aim of this study was to determine if expression of cold-inducible proteins is induced in hibernating brown bear skeletal muscle and to examine the regulation of muscle oxidant/antioxidant balance and oxidative stress.

## 2. Materials and Methods

### 2.1. Bear Sample Collection

Twenty free-ranging brown bears (*Ursus arctos*; 12 females and 8 males) were followed in Dalarna and Gävleborg counties, Sweden, from 2013 to 2017. When reaching the age of two (14 bears) or three (6 bears), the animals were captured during hibernation (February) and again during the following summer-active period (June). Immobilization was performed as previously described [56,57], and biopsies of the vastus lateralis skeletal muscle were quickly collected and immediately frozen on dry ice until storage at –80 °C. After surgery, bears were weighed and their mean body mass was found to be higher (paired student *t*-test, *p* = 0.00002) during hibernation (49.3 ± 3.0 kg) versus the summer-active period (42.2 ± 2.6 kg). Number of bears per analyses is quite variable. This is due to the variable size of biopsies collected across years, which depends on the anesthesia tolerance and the rapidity at which the veterinarians decide to work to ensure safety of the bears. The study was approved by the Swedish Ethical Committee on Animal Experiment (applications #C212/9, #C47/9, #C7/12, #C268/12, and #C18/15), the Swedish Environmental Protection Agency (NV-0758-14), and the Swedish Board of Agriculture (31-11102/12). All procedures complied with Swedish laws and regulations.

### 2.2. Proteomics Analysis of Bear Skeletal Muscles

We recently analyzed the vastus lateralis proteome for seven of the twenty bears used in the present study [58]. Here we make use of proteomics data related to oxidative stress that were not detailed by Chazarin et al. [58]. The mass spectrometry proteomics data have been deposited to the ProteomeXchange Consortium via the PRIDE [59] partner repository with the dataset identifiers PXD004908 (MS-based strategy) and PXD011687 (Gel-based strategy).

Samples from these seven bears were also used for measurement of oxidative damages (MDA-protein adducts, protein carbonyls, and 3-nitrotyrosine levels), and samples from the 13 other bears were used to complement the data using other methods (see below).

### 2.3. Quantitative RT-PCR Analyses in Bear Skeletal Muscle

Total RNA was isolated from bear muscle (N = 8 per season) using TRIzol reagent (Invitrogen, Courtaboeuf, France) according to manufacturer’s instructions. First-strand cDNAs were synthesized from 1 µg of total RNAs using PrimeScript RT kit (Ozyme, Saint Quentin en Yveline, France) with a mixture of random hexamers and oligo(dT) primers and treated with 60 units of RnaseH (Ozyme). Real-time PCR assays were performed with Rotor-Gene 6000 (Qiagen, Courtaboeuf, France). Different primers were used for cold-inducible RNA-binding protein (*CIRBP*; forward: 5′-CTGCTCAAGATCGTCCTTCC-3′; reverse: 5′-AGTCTAGTAACGAGGCCATC-3′), RNA-binding protein 3 (*RBM3*; forward: 5′-TGGTCGCAGCTACTCTAGAG-3′; reverse: 5′-CTCTGTAATTTCCTCCTGAG-3′), superoxide dismutase 1 (*SOD1*; forward: 5′-TGAAGAGAGGCATGTTGGAG-3′; reverse: 5′-CCACCTTTGCCCAAGTCATC-3′), glutathione peroxidase 4 (*GPX4*; forward: 5′-TTTACGGATCCTGGCCTTCC-3′; reverse: 5′-CTTGGGCTGGACTTTCATCCE-3′), and uncoupling protein 3 (*UCP3*; forward: 5′-CAATGGATGCCTACAGGACC-3′; reverse: 5′-CATGGATCAACAACTTCAGC-3′). The results were normalized to mRNA levels of TATA box binding protein (*TBP*; forward: 5′- AGACCATTGCACTTCGTGCC-3′; reverse: 5′-CCTGTGCACACCATTTTCCC-3′) used as a reference gene in each sample.

### 2.4. Western-Blot Analyses in Bear Skeletal Muscle

Proteins were extracted from bear muscle (N = 7 per season for NRF2 and N = 12 per season for other proteins) using ice-cold lysis buffer (Tris-HCl 20 mM, NaCl 138 mM, KCl 2.7 mM, MgCl_2_ 1 mM, glycerol 5%, NP 40 1%, EDTA 5 mM, Na_3_VO_4_ 1 mM, NaF 20 mM, and DTT 1 mM) supplemented with protease inhibitor cocktail (Sigma Aldrich, St Louis, MO, USA) as previously described [58]. Western blotting was performed as previously described [60], by separating 20 µg of total protein into each well of precast gels (Mini-protean TGX Stain-free^TM^ gel, Bio-Rad, Hercules, CA, USA). After electrophoresis, gels were UV exposed for 3 min and imaged using the Bio-Rad Chemidoc^TM^ system for quantification of protein loading. After semi-dry transfer, all membranes were blocked with 4% BSA (Bovine Serum Albumin, Euromedex, Souffelweyersheim, France) before incubation with primary antibodies. Primary antibodies were purchased from Abcam (rabbit anti-AKR7A2 [aflatoxin B1 aldehyde reductase member 2]: #ab155528; rabbit anti-CBR1 [carbonyl reductase [NADPH] 1]: #ab174852; rabbit anti-ERP29 [endoplasmic reticulum resident protein 29]: #ab11420; used at 1/1000), and Santa Cruz Biotechnology (mouse anti-NRF2 [nuclear factor, erythroid 2 like 2, also termed NFE2L2]: #sc365949; rabbit anti-catalase: #sc50508; mouse anti-HSP90AB1 [heat shock protein 90 alpha family class B member 1]: #sc-13119; mouse anti-HSPA9 [mitochondrial stress-70 protein, also termed GRP75]: #sc-133137; used at 1/1000; goat anti-β-actin: #sc-1615; used at 1/500). Corresponding secondary HRP antibodies were used for chemiluminescence visualization (Chemidoc; Bio-Rad, Hercules, CA, USA).

### 2.5. Enzyme Activity Assays in Bear Skeletal Muscle

Catalase (CAT) and glutathione peroxidase (GPX) activities were quantified in bear skeletal muscle (N = 5 per season) using commercial assay kits (BioAssay Systems, #ECAT-100 and #EGPX-100, respectively) purchased from Euromedex (Souffelweyersheim, France).

### 2.6. Reduced and Oxidized Glutathione Assays in Bear Skeletal Muscle

The content of glutathione was measured in bear skeletal muscle (N = 5 per season) using the DetectX^®^ glutathione fluorescent detection kit (Arbor Assays, Ann Arbor, MI, USA).

### 2.7. Oxidative Damage Measurements in Bear Skeletal Muscle

The content in 3-nitrotyrosine, protein carbonyls, and malondialdehyde (MDA)-protein adducts, was measured in bear skeletal muscle (N = 7 per season) using commercial ELISA kits (#STA-305, #STA-315, and #STA-332, respectively) from Cell Biolabs (San Diego, CA, USA). A commercial ELISA kit (StressMarq Biosciences Inc., #SKT-120-96S) purchased from Euromedex (Souffelweyersheim, France) was used for the quantification of 8-hydroxy-2-deoxy Guanosine (8-OHdG) in DNA extracted (DNeasy DNA; QIAGEN, Hilden, Germany) from bear skeletal muscle (N = 5 per season).

### 2.8. Statistical Analysis

Statistical analysis was performed using the R software environment v3.4.0 [61]. For all of the measurements, hibernation was compared to the summer-active period using paired student *t*-tests. Significance was set to *p*-values < 0.05.

## 3. Results

### 3.1. Gene Expression of Cold-Inducible Proteins and Uncoupling Protein 3 in Bear Skeletal Muscle

**Figure 1:**
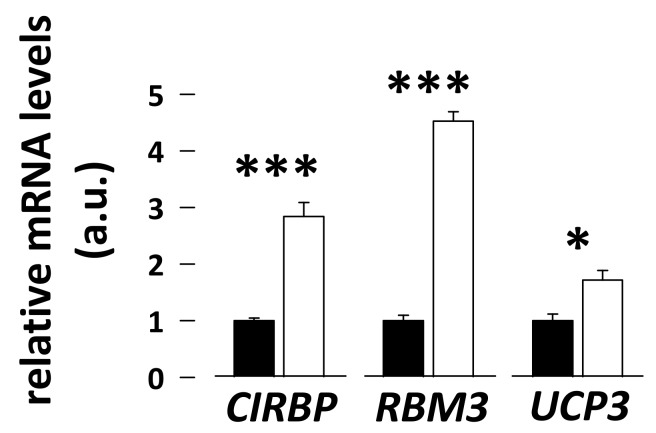
Gene expression of cold-inducible proteins and uncoupling protein 3 (UCP3) in bear skeletal muscle. Levels of cold-inducible RNA-binding protein (*CIRBP*), RNA-binding protein 3 (*RBM3*), and *UCP3* were measured using RT-qPCR in vastus lateralis muscle samples from summer-active (black bars) and hibernating (white bars) brown bears. Data (N = 8/group) are expressed as means ± sem, with values in the hibernating period being normalized to those in the summer-active condition, which were arbitrarily set to 1. Statistical significance is shown for paired student *t*-tests (* *p* < 0.05; *** *p* < 0.0001). a.u.: arbitrary units.

Cold-inducible *CIRBP*, *RBM3*, and *UCP3* mRNA levels were 2.8-, 4.5-, and 1.7-fold higher, respectively, in skeletal muscles of hibernating compared to summer-active bears (Figure 1; N = 8 per season; paired student *t*-test, *p* = 0.00007, *p* = 0.0000005, and *p* = 0.003, respectively).

### 3.2. Protein Abundance of Subunits of Mitochondrial Respiratory Complexes I, II, and III in Bear Skeletal Muscle

**Figure 2:**
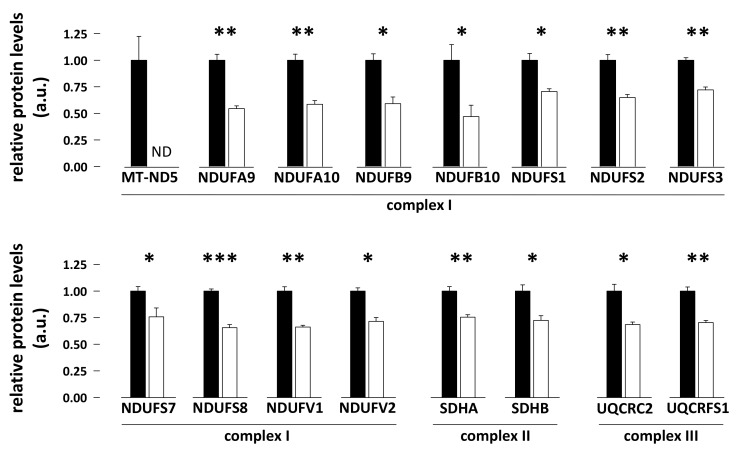
Protein abundance of subunits of mitochondrial respiratory complexes I, II, and III in bear skeletal muscle. Levels of subunits of the mitochondrial electron transfer chain (ETC) complex I, II, and III were measured using proteomics in vastus lateralis muscle samples from summer-active (black bars) and hibernating (white bars) brown bears [58]. Data (N = 7/group) are expressed as means ± sem, with values in the hibernating period being normalized to those in the summer-active condition, which were arbitrarily set to 1. Statistical significance is shown for paired student *t*-tests (* *p* < 0.05; ** *p* < 0.001; *** *p* < 0.0001). a.u.: arbitrary units; ND: below the detection threshold.

From a proteomics analysis of bear skeletal muscle (vastus lateralis) that we recently published [58], we extracted here detailed data about protein expression levels of subunits of mitochondrial respiratory complexes I, II, and III. We found that the abundance of subunits NDUFA9, NDUFA10, NDUFB9, NDUFB10, NDUFS1, NDUFS2, NDUFS3, NDUFS7, NDUFS8, NDUFV1, and NDUFV2 of complex I (NADH dehydrogenase) was generally 1.4–2.1-fold lower in skeletal muscle of hibernating compared to summer-active bears (Figure 2; N = 7 per season; paired student *t*-tests, *p* = 0.00042, *p* = 0.00023, *p* = 0.0059, *p* = 0.035, *p* = 0.0015, *p* = 0.00035, *p* = 0.00066, *p* = 0.046, *p* = 0.000015, *p* = 0.00013, and *p* = 0.0012, respectively). Subunit MT-ND5 of complex I was not detectable in the skeletal muscle of hibernating bears. Only NDUFA8, NDUFB6, and NDUFS4 levels remained unchanged in hibernating versus summer-active bears (N = 7 per season; paired student *t*-tests, *p* = 0.68, *p* = 0.61, and *p* = 0.33, respectively). Concerning complex II (succinate dehydrogenase), subunits SDHA and SDHB were significantly 1.3–1.4-fold less abundant in skeletal muscle of hibernating compared to summer-active bears (Figure 2; N = 7 per season; paired student *t*-tests, *p* = 0.00078 and *p* = 0.014, respectively). Finally, the levels of subunits UQCRC2 and UQCRFS1 of complex III (ubiquinol-cytochrome c reductase) were 1.4-fold lower in skeletal muscle of hibernating compared to summer-active bears (Figure 2; N = 7 per season; paired student *t*-tests, *p* = 0.0011 and *p* = 0.00095, respectively). Subunit UQCRC1 of complex III was also detected and it was observed that its levels remained stable (N = 7 per season; paired student *t*-tests, *p* = 0.19) between hibernation and the summer-active period.

### 3.3. Expression Levels and Activity of Antioxidant Systems in Bear Skeletal Muscle

**Figure 3:**
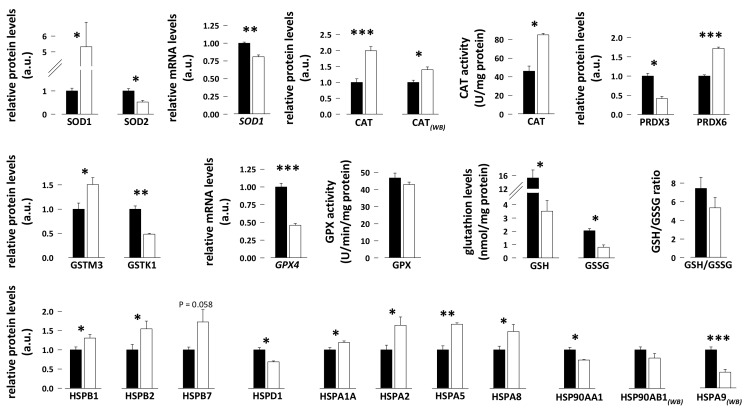
mRNA/protein levels and/or activity of antioxidant systems in bear skeletal muscle. Levels of antioxidant enzymes and heat shock proteins (HSPs) were measured using proteomics [58], and/or western blotting (WB; see also Figure S1), and/or RT-qPCR (mRNA) in vastus lateralis muscle samples from summer-active (black bars) and hibernating (white bars) brown bears. Enzyme activities and the levels of reduced (GSH) and oxidized (GSSG) glutathione were measured using commercial kits (see the text). Data (N = 5–12/group) are expressed as means ± sem, with values in the hibernating period being normalized to those in the summer-active condition, which were arbitrarily set to 1. Statistical significance is shown for paired student *t*-tests (* *p* < 0.05; ** *p* < 0.001; *** *p* < 0.0001). a.u.: arbitrary units.

Among antioxidant enzymes, we observed that protein abundance of peroxiredoxin-3 (PRDX3) was 2.4-fold lower in skeletal muscle of hibernating compared to summer-active bears (Figure 3; N = 7 per season; paired student *t*-tests, *p* = 0.0023). The opposite was observed for peroxiredoxin-6 (PRDX6) whose abundance was 1.7-fold higher during hibernation (Figure 3; N = 7 per season; paired student *t*-tests, *p* = 0.00000045). Skeletal muscle levels of two other peroxiredoxins, PRDX1 and PRDX2, remained unchanged between hibernating and summer-active animals (N = 7 per season; paired student *t*-tests, *p* = 0.98 and *p* = 0.15, respectively). The mRNA levels of superoxide dismutase [Cu-Zn] (SOD1) were 1.2-fold lower in skeletal muscle of hibernating compared to summer-active bears (Figure 3; N = 8 per season; paired student *t*-test, *p* = 0.00023). Conversely, SOD1 protein levels were 5.3-fold higher in skeletal muscle of hibernating bears (Figure 3; N = 7 per season; paired student *t*-test, *p* = 0.03). The levels of mitochondrial superoxide dismutase [Mn] (SOD2) were reduced by half in the hibernating muscle (Figure 3; N = 7 per season; paired student *t*-test, *p* = 0.026). In the hibernation state, a 1.4–2-fold increase in catalase (CAT) protein levels (Figure 3; N = 7 per season for proteomics data and N = 6 for western blot analysis; paired student *t*-tests, *p* = 0.00000063 and *p* = 0.0015, respectively) was observed and CAT activity was also approx. doubled (Figure 3; N = 5 per season; paired student *t*-test, *p* = 0.0022).

We also explored the glutathione system and observed that glutathione S-transferase Mu 3 (GSTM3) protein levels were 1.5 times increased in skeletal muscle of hibernating compared to summer-active bears (Figure 3; N = 7 per season; paired student *t*-test, *p* = 0.0026), whereas the protein levels of glutathione S-transferase kappa 1 (GSTK1) were decreased by half at the same time (Figure 3; N = 7 per season; paired student *t*-test, *p* = 0.00021) and the protein levels of glutathione S-transferase P (GSTP1), glutathione S-transferase Mu 1 (GSTM1), and glutathione peroxidase 1 (GPX1) remained unchanged (N = 7 per season; paired student *t*-tests, *p* = 0.89, *p* = 0.11, and *p* = 0.84, respectively). The mRNA levels of glutathione peroxidase 4 (*GPX4*) were twofold lower in skeletal muscle of hibernating compared to summer-active bears (Figure 3; N = 8 per season; paired student *t*-tests, *p* = 0.00007). Glutathione peroxidase (GPX) activity was not significantly different when comparing the hibernation versus summer-active states (N = 5 per season; paired student *t*-tests, *p* = 0.54). The muscle content of both reduced (GSH) and oxidized (GSSG) forms of glutathione were reduced during hibernation, by a factor of 4.3 and 2.5, respectively (Figure 3; N = 5 per season; paired student *t*-tests, *p* = 0.024 and *p* = 0.014, respectively), resulting in a slight 1.4-fold lower GSH to GSSG ratio in hibernating bears (Figure 3; N = 5 per season; paired student *t*-test, *p* = 0.29).

Heat shock proteins are stress-responsive proteins. Their protein levels were generally induced (1.2–1.7 times) in bear skeletal muscle during hibernation, including for heat shock proteins beta-1 (HSPB1), beta-2 (HSPB2), and beta-7 (HSPB7), heat shock 70 kDa proteins 1A (HSPA1A) and 2 (HSPA2), 78 kDa glucose-regulated protein (HSPA5), and heat shock cognate 71 kDa protein (HSPA8) (Figure 3; N = 7 per season; paired student *t*-tests, *p* = 0.002, *p* = 0.0011, *p* = 0.059, *p* = 0.029, *p* = 0.043, *p* = 0.00049, and *p* = 0.042, respectively). On the reverse, muscle levels of mitochondrial 60 kDa heat shock protein (HSPD1), heat shock protein HSP 90-alpha (HSP90AA1), and mitochondrial stress-70 protein (HSPA9 or GRP75) were 1.4–2.4-fold lower during hibernation (Figure 3; N = 7 per season for proteomics data and N = 12 for western blot analysis; paired student *t*-tests, *p* = 0.002, *p* = 0.0078, *p* = 0.000071, respectively), and those of heat shock protein HSP 90-beta (HSP90AB1) and heat shock protein beta-6 (HSPB6) remained unchanged (N = 7 per season; paired student *t*-tests, *p* = 0.20, and *p* = 0.11, respectively.

### 3.4. Levels of Oxidative Damages in Bear Skeletal Muscle

**Figure 4:**
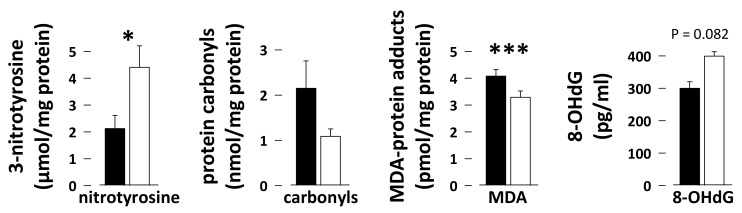
Oxidative damages in bear skeletal muscle. Levels of 3-nitrotyrosine, protein carbonyls, malondialdehyde (MDA)-protein adducts, and 8-hydroxy-2-deoxy Guanosine (8-OHdG) were measured using commercial ELISA kits in vastus lateralis muscle samples from summer-active (black bars) and hibernating (white bars) brown bears. Data (N = 5–7/group) are expressed as means ± sem. Statistical significance is shown for paired student *t*-tests (* *p* < 0.05; *** *p* < 0.0001).

The vastus lateralis content in 3-nitrotyrosine was twofold higher in skeletal muscles of hibernating compared to summer-active bears (Figure 4; N = 7 per season; paired student *t*-test, *p* = 0.018) and the content in 8-hydroxy-2-deoxy Guanosine (8-OHdG) in DNA extracted from bear skeletal muscle tended to be higher during hibernation but without reaching significance (Figure 4; N = 5 per season; paired student *t*-test, *p* = 0.082). On the contrary, the skeletal muscle levels of malondialdehyde (MDA)-protein adducts were 1.2-fold lower during hibernation (Figure 4; N = 7 per season; paired student *t*-test, *p* = 0.000071). The twofold reduction in muscle protein carbonyl levels during hibernation did not reached significance (N = 7 per season; paired student *t*-test, *p* = 0.17), due to inter-individual variations.

### 3.5. Protein Abundance of Nuclear Factor E2-Related Factor 2 (NRF2) and Selected Downstream Targets in Bear Skeletal Muscle

**Figure 5:**
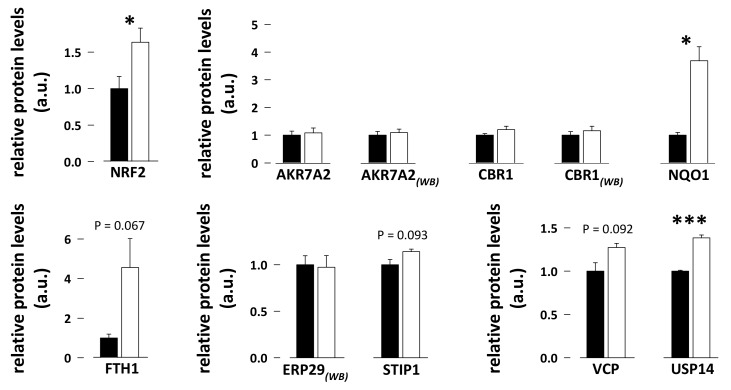
Protein levels of nuclear factor E2-related factor 2 (NRF2) and selected downstream targets in bear skeletal muscle. Levels of proteins of the NRF2 pathway were measured using proteomics [58] and/or western blotting (WB; see also Figure S1) in vastus lateralis muscle samples from summer-active (black bars) and hibernating (white bars) brown bears. Data (N = 5–12/group) are expressed as means ± sem, with values in the hibernating period being normalized to those in the summer-active condition, which were arbitrarily set to 1. Statistical significance is shown for paired student *t*-tests (* *p* < 0.05; *** *p* < 0.0001). a.u.: arbitrary units.

Abundance of nuclear factor E2-related factor 2 (NRF2 or NFE2L2) and of NAD(P)H dehydrogenase [quinone] 1 (NQO1) and ubiquitin carboxyl-terminal hydrolase 14 (USP14) was 1.6-fold, 3.7-fold, and 1.4-fold higher, respectively, in skeletal muscle of hibernating compared to summer-active bears (Figure 5; N = 7 per season; paired student *t*-tests, *p* = 0.012, *p* = 0.0024, and *p* = 0.000027, respectively). Ferritin heavy chain (FTH1) exhibited increased levels (4.6-fold) during hibernation, but without reaching significance (N = 7 per season; paired student *t*-test, *p* = 0.067). Finally, no difference was found when comparing the hibernation and summer-active states for muscle aflatoxin B1 aldehyde reductase member 2 (AKR7A2), carbonyl reductase [NADPH] 1 (CBR1), endoplasmic reticulum resident protein 29 (ERP29), stress-induced-phosphoprotein 1 (STIP1), and transitional endoplasmic reticulum ATPase (VCP) (N = 7 per season; paired student *t*-tests, *p* = 0.32, *p* = 0.22, *p* = 0.79, *p* = 0.094, and *p* = 0.092, respectively).

## 4. Discussion

During hibernation, bears demonstrate resistance to muscle atrophy over prolonged periods of physical inactivity and fasting [41,42]. This may involve a specific molecular proteomic signature of skeletal muscle during hibernation and the role of n-3 polyunsaturated fatty acids like docosahexaenoic acid [58], as well as existence of circulating antiproteolytic components in hibernating bears [60]. The present study explored the regulation of muscle cold-inducible proteins, the oxidant/antioxidant balance, and oxidative stress as another mechanism possibly contributing to muscle preservation in hibernating bears. The results show that, consistently with the increased expression of cold-inducible proteins and antioxidant systems, limited oxidative stress may favor bear skeletal muscle maintenance during months of hibernation.

### 4.1. Induction of Cold-Inducible Protein Expression During Hibernation in Bear Skeletal Muscle

We show that both *RBM3* and *CIRBP* mRNA levels are higher in skeletal muscle of brown bears during hibernation (Figure 1). This appears to be a feature common to a number of hibernators. Indeed, the mRNA levels of *RBM3* have been shown to increase in brain, heart, and liver tissues of hibernating golden-mantled ground squirrel (*Spermophilus lateralis*) [62], in brown adipose tissue, liver, heart, skeletal muscle, and hypothalamus of hibernating S. *parryii* [63], and in muscle, liver, and heart tissues of hibernating *U. americanus* [49,64]. Increased levels of a short form of CIRBP, encoding the full-length protein, have also been reported in the heart of hibernating hamsters [65].

In rodents, overexpression of RBM3 has been shown to trigger skeletal muscle hypertrophy and attenuate atrophy as well in vitro as in vivo [66]. Such effects could be linked to previously proposed mechanisms for RBM3 action, such as enhancement of global protein synthesis at both 37 °C and 32 °C [67], and reduction of apoptosis [55]. CIRBP has also been shown to exert anti-apoptotic effects at 32 °C [45]. The induction of cold-inducible protein expression in skeletal muscle of hibernating bears could then act to prevent an exaggerated decrease in protein synthesis and/or inhibit apoptosis, thereby favoring muscle maintenance.

Cold-inducible proteins have also been involved in quenching ROS and alleviating oxidative stress [50,51,52,53,54,55]. Therefore, we hypothesize that the oxidant/antioxidant balance is regulated toward prevention of oxidative stress in skeletal muscle of hibernating brown bears.

### 4.2. Downregulation of Mitochondrial Electron Transfer Chain (ETC) Complexes I, II, and III May Favor Reduced Production of ROS during Hibernation in Bear Skeletal muscle

Mitochondria are an important source of ROS within most mammalian cells. Of the 11 mitochondrial ROS-producing sites, the major ones are located in ETC complexes I, II, and III [68]. By extracting detailed proteomics results from a previous study [58], we observed that the abundance of 12/15 subunits of complex I, 2/2 subunits of complex II, and 2/3 subunits of complex III were significantly reduced in bear skeletal muscle during hibernation (Figure 2). As previously suggested [58], the mitochondrial content is likely decreased in hibernating bear muscle, which may contribute to the general decrease that we observed in the abundance of ETC complex subunits. In addition, we recently showed that lipid oxidation is reduced in hibernating brown bear muscle [58], which may contribute to suppress electron supply to the respiratory chain. Finally, uncoupling protein 3 (UCP3) has been attributed a role in protecting mitochondria against oxidative stress [69,70]. Therefore, the increased expression of UCP3 that we observed during hibernation (Figure 1) is not only in line with its known induction during mild hypothermia [71], but it could also constitute a mechanism to mitigate oxidative damages in the hibernating muscle. This would be in accordance with the “uncoupling to survive” hypothesis, which postulates that uncoupling proteins can decrease ROS production by lowering the potential of the inner mitochondrial membrane [72], providing that UCP3 actually reduces the mitochondrial proton motive force, a still debated question [73]. Altogether, the data suggest a reduction of mitochondrial ROS production in bear skeletal muscle during hibernation. This would be consistent with the known reduction in ROS production in skeletal muscle of hibernating *I. tridecemlineatus* [33].

### 4.3. Upregulation of Key Antioxidant Systems may Favor ROS Scavenging during Hibernation in Bear Skeletal Muscle

Endogenous muscle antioxidants include several enzymes like superoxide dismutases (SOD1 in the cytosol and SOD2 in the mitochondria), catalase (CAT), and peroxiredoxins (PRDXs), as well as the glutathione system encompassing enzymes like glutathione S-transferases (GSTs) and glutathione peroxidases (GPXs), and their cofactor, glutathione (GSH in its reduced form and GSSG in its oxidized form) [11,15]. The regulations we observed (Figure 3) suggest that the upregulation of SOD1 protein levels during hibernation is due to post-transcriptional events, which could involve microRNAs [74] or ELAV proteins [75]. This is a mechanism that could favor dismutation of cytosolic O_2_•^−^ to form H_2_O_2_, which reduction to water is likely favored by the increase in CAT and PRDX6 protein levels and the activity that we report here in the hibernating bear muscle. Consistently, increased levels of catalase [32] and a higher total antioxidant capacity [31] have been found during hibernation in muscle of *I. tridecemlineatus*. Enhanced expression of GPX, CAT, and SOD1 has also been shown in muscle of the hibernating ground squirrel *S. citellus* [34]. Because PRDX6 can reduce lipid hydroperoxides [76], its upregulation may also favor protection of the membranes of bear muscle cells during hibernation. Finally, lower levels of the mitochondrial SOD2 and PRDX3 proteins might reflect the reduction of that mitochondrial ROS production during hibernation in bears, as suggested above. This may be a feature depending on the animal species considered and/or whether interbout arousals are occurring during hibernation, as SOD2 protein levels have been reported to increase in the quadriceps muscle of hibernating *I. tridecemlineatus* [32], but decrease in the quadriceps muscle of hibernating *S. citellus* [34].

The glutathione system is of utmost importance in protecting cells against oxidative damages. GSH can directly scavenge ROS or act as a cofactor for GPXs and GSTs to reduce lipid hydroperoxides [77]. The increase in cytosolic GSTM3 might help in preventing accumulation of ROS and lipid peroxides in skeletal muscle of hibernating bears. On the other hand, the observed decrease in the mitochondria-specific GST, GSTk1, is another indication that ROS-induced peroxidation is decreased in the mitochondrial compartment of hibernating bear muscle cells. Finally, the maintenance of muscle GPX activity and the stability of the GSH-to-GSSG ratio, a classical marker of oxidative stress, suggest that bear skeletal muscle is not under a significant oxidative insult during hibernation.

Apart from their role as molecular chaperones, heat shock proteins (HSPs) have been involved in protection against oxidative stress [78,79]. HSPs exert their protective effects by sensing redox changes, repairing or removing damaged proteins, favoring the action of antioxidants, and also via anti-apoptotic effects [78]. A specific role against oxidative stress has notably been reported for HSPB1 [80] and members of the HSP70 subfamily [81]. The induction of HSP expression that we observed in skeletal muscle of hibernating bears concerns the small HSPs (HSPB1 or HSP27, HSPB2, HSPB7, and HSPB6) and HSP70 (HSPA1A, HSPA2, HSPA5 or GRP78, and HSPA8) subfamilies. This may provide a protective effect against H_2_O_2_ and accumulation of damaged/misfolded proteins. These results extend previous findings showing elevated levels of phosphorylated HSPB1 and of HSP70 in skeletal muscle of bats during hibernation [25,82]. HSPA9 or GRP75 is mainly located in the mitochondria. As already suggested for the regulation of other mitochondrial proteins (see above), its decreased expression observed here in hibernating bears is an additional element supporting the view that mitochondrial oxidative stress does not occur in skeletal muscle during hibernation. The situation appears to be different in mitochondria from hibernating ground squirrels, with for example HSPA9 being overexpressed in muscle of hibernating *I. tridecemlineatus* [1].

### 4.4. Oxidative Damages during Hibernation in Bear Skeletal Muscle

Adverse effects of oxidative stress include oxidative damages to lipids, proteins, and DNA [10]. The free radical •OH can for example cause lipid peroxidation, which leads to formation of malondialdehyde (MDA)-protein adducts. Oxidation of proteins due to ROS notably produces the so-called protein carbonyl derivatives, and O_2_•^−^ and nitric oxide (•NO) together or nitrogen dioxide (•NO_2_) alone can lead to the modification of protein tyrosine residues to 3-nitrotyrosine. Finally, oxidative lesions of DNA are also triggered in response to oxidative stress, with the formation of, e.g., 8-hydroxy-2-deoxy Guanosine (8-OHdG). The reduced levels of MDA-protein adducts and of protein carbonyls (twofold, not significant because of inter-individual variations), and the unchanged levels of 8-OHdG that we observed in muscle of hibernating versus summer-active bears altogether argue for a drastic reduction of muscle oxidative stress during hibernation. Consistent results have been obtained in hibernating *I. tridecemlineatus*, with no evidence for muscle protein oxidation (carbonyl levels) [32]. Only the levels of 3-nitrotyrosine-containing proteins were observed to be increased in hibernating bear skeletal muscle. As our results suggest a reduction of ROS-induced damage, this may reflect an increase in nitrosative stress in hibernating bear muscle, which should be explored in detail in future studies. Endogenous production of •NO and •NO_2_ largely relies on arginine availability [83]. It could be that low levels of protein degradation during hibernation nevertheless make muscle arginine available enough to increase production of •NO and •NO_2_. Mitochondrial respiration is inhibited by the action of •NO [84], which could be a mechanism whereby metabolic suppression is achieved in bear muscle cells.

### 4.5. NRF2 Regulation during Hibernation in Bear Skeletal Muscle

Nuclear factor, erythroid 2-like 2 (NFE2L2 or NRF2) is a transcription factor that is well known to control the response to oxidative stress by notably regulating the expression of antioxidant and detoxification enzymes [85,86]. A key role has already been attributed to NRF2 in regulating antioxidant defences in hibernating *I. tridecemlineatus* [87] and *S. dauricus* [23]. Such a role for NRF2 appears also valid for hibernating bears, as increased levels of antioxidant enzymes (see above) were paralleled by the enhanced expression of NRF2 in skeletal muscle during hibernation (Figure 5). Among NRF2 targets, apart from those presented in Figure 3, we also found an increased expression of the ferritin heavy chain (FTH1), an antioxidant protein, of NAD(P)H dehydrogenase [quinone] 1 (NQO1), a detoxification enzyme, and of ubiquitin carboxyl-terminal hydrolase 14 (USP14), which is involved in the degradation of damaged proteins. However, no change was observed due to hibernation for few other detoxification enzymes (AKR7A2, CBR1) and chaperones (ERP29 and STIP1). This may indicate a complex scheme for NRF2 action, which could selectively regulate antioxidant enzymes but no other targets in skeletal muscle of hibernating bears. To deepen these results on NRF2, the phosphorylated status of NRF2, and the possible involvement of the E3 ubiquitin ligase KEAP1 (Kelch ECH-associating protein 1) and small MAF proteins should be studied. However, we did not succeed in detecting these important factors. Alternatively, it is also likely that other transcription factors and/or pathways may play a role in regulating the response to oxidative stress in bear skeletal muscle during hibernation. Indeed, transcription factors other than NRF2, like for example nuclear factor κB, are known to control the expression of antioxidant genes [75]. Moreover, as already stated above for SOD1, microRNAs could be involved in the regulation of antioxidant enzyme expression, a field that largely remains to be investigated.

## 5. Conclusions

**Figure 6:**
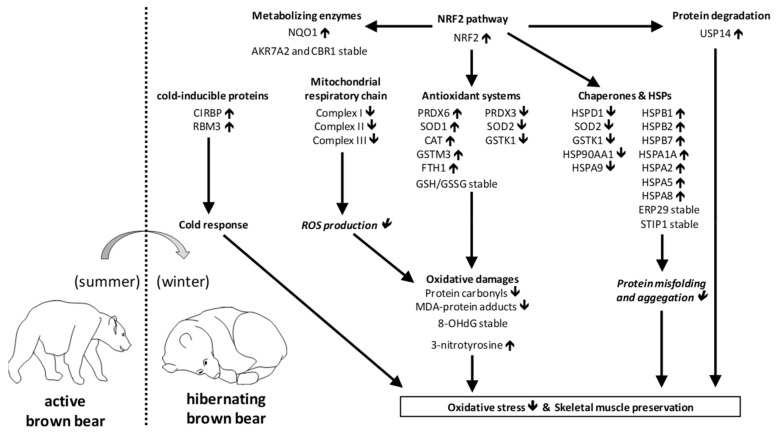
Proposed mechanisms based on reduced oxidative stress promoting skeletal muscle preservation in hibernating versus summer-active brown bears. In italic are shown features that were not directly assessed.

During hibernation, the response to skeletal muscle oxidative stress has been partly studied, however only in a fragmented fashion in several studies of ground squirrels (see above). Here we examined for the first time the comprehensive aspects of the regulation of oxidative stress and its possible influence on muscle preservation in hibernating bears. In line with the reduced levels of oxidative damages, the specific response to mild hypothermia (cold response), decreased production of ROS due to metabolic suppression, increased scavenging of ROS due to the induction of antioxidant systems, and reduced accumulation of misfolded proteins due to the induction of chaperones and HSPs, appear as key mechanisms to decrease oxidative stress and promote skeletal muscle preservation during brown bear (*U. arctos*) hibernation (Figure 6). Beyond the hibernation period, the antioxidant protection may also prevent an increase of the muscle oxidative insult at hibernation exit, due to the restoration of metabolic rates and mitochondrial oxygen consumption. Our findings showing how the oxidant-antioxidant balance is regulated in hibernating bears may provide specific targets of therapeutic interest to improve health outcomes related to muscle atrophy during sarcopenia, muscle disuse or microgravity conditions.

Future studies should specifically measure the levels of ROS and reactive nitrogen species in hibernating bear muscle. Because ROS play a role in several signaling pathways [88], the impact of any change in ROS levels on cellular signaling (e.g., NF-κB, MAPKs, and PI3K-Akt signaling pathways) should be evaluated during hibernation. The influence of transcription factors other than NRF2 on antioxidant systems expression would also bring a more comprehensive understanding of the mechanisms controlling cellular oxidative homeostasis.

## Supplementary Materials

The following are available online at https://www.mdpi.com/2076-3921/8/9/334/s1, Figure S1: Representative blots of bear skeletal muscle proteins.
