# Supplementary material for: Limited Oxidative Stress Favors Resistance to Skeletal Muscle Atrophy in Hibernating Brown Bears (Ursus Arctos)
Source: Antioxidants (Basel). 2019 Aug 22;8(9):334. doi: 10.3390/antiox8090334 (PMC6770786; doi:10.3390/antiox8090334)
Supplement: Supplementary file 1 [file antioxidants-08-00334-s001.pdf]

Limited oxidative stress favors resistance to skeletal muscle atrophy in hibernating brown bears (*Ursus arctos*)

Blandine Chazarin, Anna Ziemianin, Alina L. Evans, Emmanuelle Meugnier, Emmanuelle Loizon, Isabelle Chery, Jon M. Arnemo, Jon E. Swenson, Guillemette Gauquelin-Koch, Chantal Simon, Stephane Blanc, Etienne Lefai, and Fabrice Bertile*

* Corresponding author: Phone: +33 3 68 85 26 81; Email address: fbertile@unistra.fr; Université de Strasbourg, CNRS, IPHC UMR 7178, F-670000 Strasbourg, France

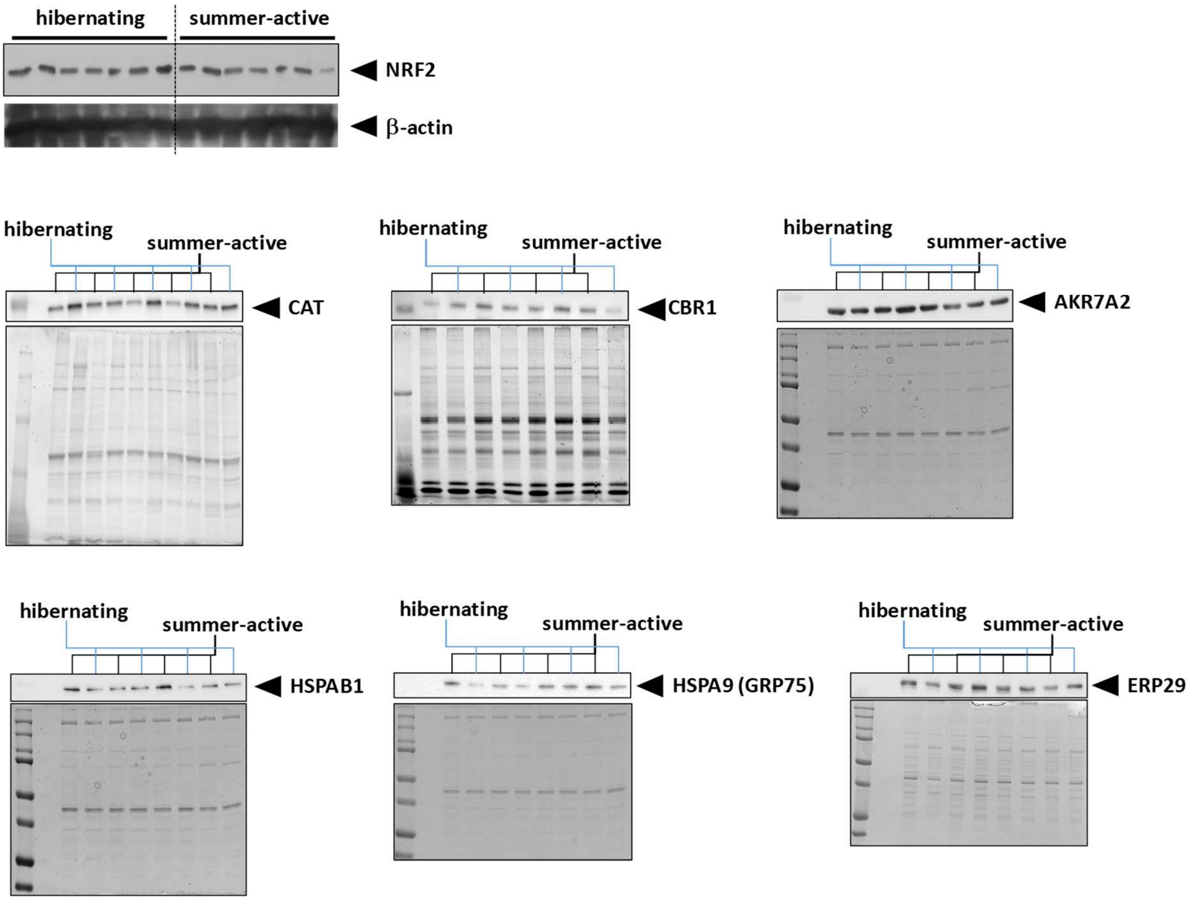

Supplementary Figure S1. Representative blots of bear skeletal muscle proteins. Shown are representative images that have been obtained through western-blot analysis of the levels of catalase (CAT), heat shock protein HSP 90-beta (HSPAB1) mitochondrial stress-70 protein (HSPA9 or GRP75), nuclear factor E2-related factor 2 (NRF2 or NFE2L2), aflatoxin B1 aldehyde reductase member 2 (AKR7A2), carbonyl reductase (CBR1), and endoplasmic reticulum resident protein 29 (ERP29) in bear vastus lateralis muscles (N = 12/ group, except from NRF2: N = 7/ season). Corresponding quantifications can be seen in Figures 3 and 5.
